# Systemic Lupus Erythematosus in a 12-Year-Old Boy: A Rare Entity in Childhood

**DOI:** 10.5005/jp-journals-10005-1077i

**Published:** 2010-09-15

**Authors:** Shilpashree HS

**Affiliations:** 1 Senior Lecturer, Department of Oral Medicine and Radiology, Seema Dental College and Hospital, Rishikesh, Uttarakhand, India

**Keywords:** SLE, Malar rash, Oral ulcers, Children.

## Abstract

Childhood onsets of systemic lupus erythematosus (SLE) are quite rare and comprise less than 20% of all cases of lupus. Prepubertal onset is even rarer, as most children with SLE belong to the adolescent age group. SLE is an immune complex mediated disease with multiple organ system involvement, which may lead to high mortality and morbidity. We report a 12-year-old boy with SLE who presented clinically with malar rashes, joint pain and oral ulcers.

## INTRODUCTION

Systemic lupus erythematosus (SLE) is characterized by the production of autoantibodies and immune complexes leading to protean systemic manifestations.^1^ The clinical manifestations of SLE vary widely in affecting systems and organs and the course of disease fluctuates between periods of exacerbations and remission. This chronic inflammatory disease is named from its characteristic erythematous patches crossing the bridge of the nose forming a butterfly configuration on the malar regions of the face. The term “LUPUS” is derived from erosive nature of condition, which was likened to the damage wrought by a hungry wolf.^2^ Though the disease is widely described in adolescents and adults, literature pertaining to childhood SLE in India is limited. Childhood and prepubertal onset occurs in less than 20% of all cases.^3,4^ Some of the common clinical abnormalities in patients with SLE in terms of percentage are as follows.^5^

1. Discoid facial lesions―20%
2. Bacterial infections―40%
3. Lymphadenopathy―50%
4. Alopecia―50%
5. Butterfly rash on face―60%
6. Anemia―70%
7. Renal involvement―75%
8. Fatigue and fever―90%

Diagnosis is mainly based on presence of autoantibodies.^1^

## CASE REPORT

The patient, a 12-year-old boy, came to Department of Oral Medicine and Radiology with the complaint of spacing between upper front teeth. On examination, the patient was in ugly duckling stage of dentition and extraoral malar rash over the zygomatic bone crossing over the bridge of the nose.

Patient gave history of malar rash since 8 months, which initially was of small sized spots and then gradually increased and extended across the bridge of nose and became erythematous on exposure to sun. He applied ointment on rashes and discontinued due to lack of improvement. He also gave history ofjoint pain, pain in abdomen and intermittent fever since 8 months, not associated with chills and rigors. Patient also gave history of chest pain on inspiration since 1 month.

**Fig. 1:**
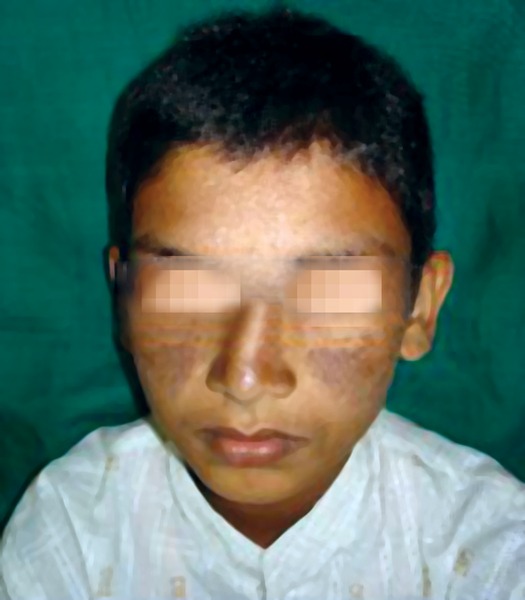
Extraoral features showing butterfly shaped malar rashes

**Fig. 2:**
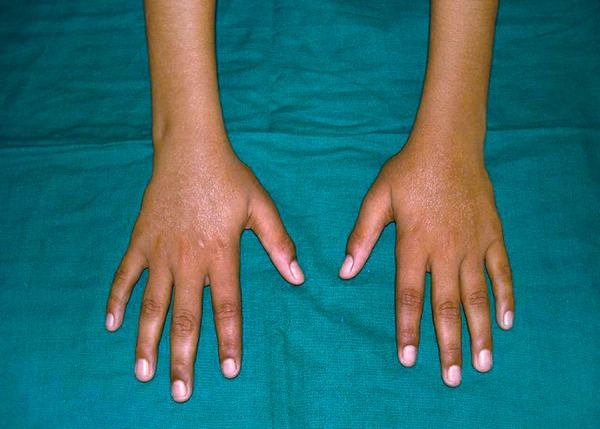
Localized hypopigmented areas over dorsal surface of hands

**Fig. 3:**
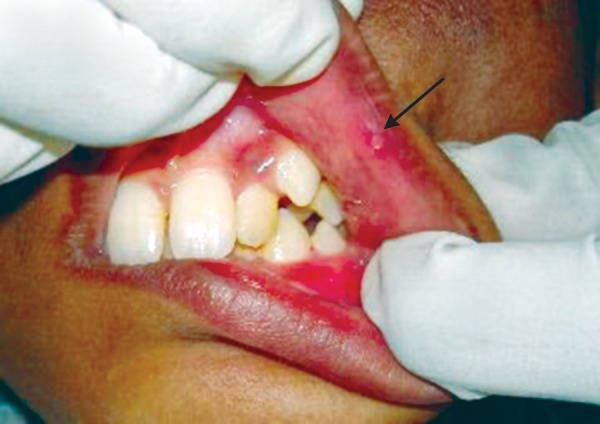
Ulcers on the vermilion border of the upper lip

**Fig. 4:**
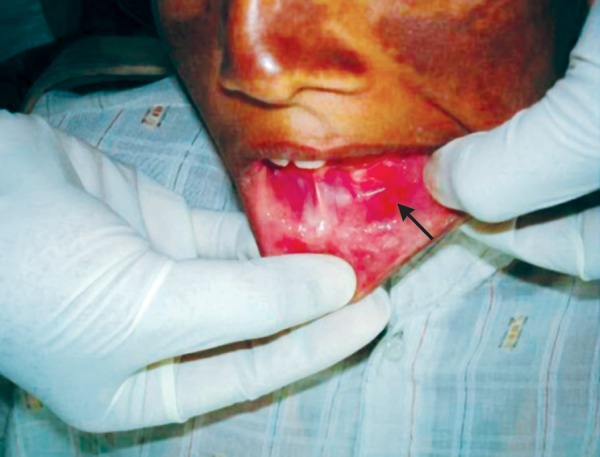
Ulcers surrounded by erythematous halo on lower lip

Extraoral examination of patient revealed maculopapular rash in the malar areas, which extended across the bridge of nose and some parts of forehead (Fig. 1). Examination of hands revealed localized hypopigmented areas over dorsal surface (Fig. 2). Intraoral examination revealed ulcers, one on vermilion border of the upper lip measuring 2 mm and one on the lower labial mucosa measuring 3 mm in diameter. Ulcers were surrounded by erythematous halo and floor was yellowish (Figs 3 and 4).

**Fig. 5:**
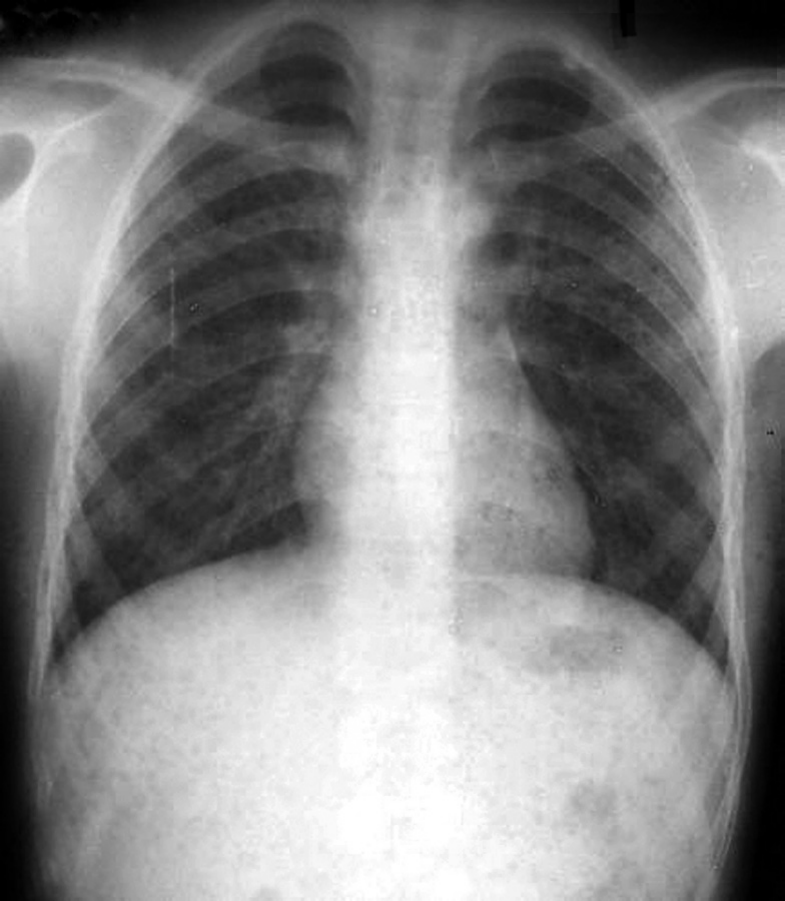
Chest radiograph of patient

**Fig. 6:**
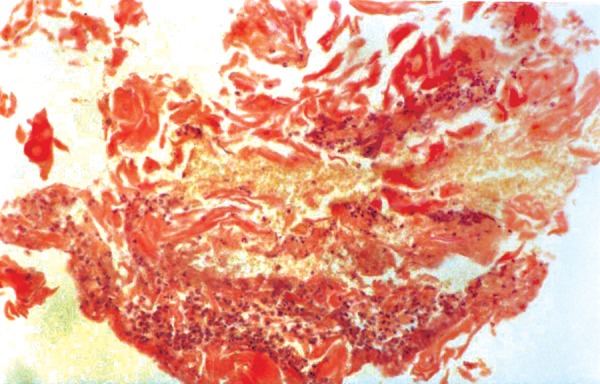
Histological picture showing characteristic features of SLE

After considering history and the clinical features, patient was subjected to the investigations like blood chemistry, blood counts, chest radiograph (Fig. 5), ECG, echocardiography all of which were normal. But, antinuclear antibodies (ANA) test and LE cell phenomenon were positive. Histological examination of skin biopsy specimen revealed hyperkeratosis, keratin plugging, atrophy of rete pegs and focal hydropic degeneration of basal cells. Dermis exhibited degenerating collagen fibers, hyalinization and focal pigmentation (Fig. 6).

After considering the clinical features, ANA test, LE cell phenomenon and histopathological findings, the diagnosis of systemic lupus erythematosus was made.

In treatment plan, patient was advised to avoid sun exposure and treated with topical application of steroids for malar rashes and symptomatic treatment of oral ulcers. The patient was kept under the periodic observations.

## DISCUSSION

SLE is a multisystem disease with a wide variety of clinical manifestations. SLE is highly prevalent in females. The female-male ratio is 5:1 in children, 9:1 in adults and 3:1 in old age people. But, drug induced SLE shows no sex predilection. Idiopathic SLE occurs between first and fourth decade. SLE occurs in all races. In United States, the average incidence is 25.5 per million in white females and two to three times higher in African-American and Hispanic females. The specific etiology of SLE is not known exactly, but immunocomplexes, autoantibodies, genetic, infections, drugs, environmental and endocrine factors play a significant role.^6^

SLE arise from aberrant immune behavior, the “autoimmune” is used to describe the reaction of person’s own antibodies developed against his or her own tissues.^1^ SLE is preceded by complicated autoimmune changes. Anti- nuclear, anti-Ro, anti-La and antiphospholipid antibodies appear first, followed by anti-double-stranded DNA antibodies, and then by anti-Sm and antinuclear ribonucleoprotein antibodies. The number of autoantibody types continues to increase until the time of diagnosis and therapeutic intervention.^7^ These different antibodies may be formed by antibody forming B lymphocytes or by stimulation of antigens.^8^ These immune complexes set off an array of immunological reactions resulting in activation of complement system, which attracts neutrophils and macrophages. This in turn leads to vasculitis, fibrosis and tissue necrosis.^1^

Clinical patient with SLE may present with fever, weakness, fatigue, weight loss as the first symptoms. According to Biesecker et al, joint involvement is the most common manifestation of SLE.^9^ Arthritis, usually symmetric, most commonly affects proximal interphalangeal joints, knees, wrists, and metacarpophalangeal joints. Depression, anorexia, dry skin, and pruritis, along with other dermatologic problems, gastrointestinal distress, constipation, and muscle spasms are among other common complaints of SLE patients.^2^

Shahid Iqbal et al conducted a study in SLE patients and noticed that manifestations of SLE in children are diverse. A detailed history, thorough review of systems, complete physical examination, complete blood count, urinalysis, and high index of suspicion help to make the correct diagnosis of SLE in patients with atypical presentations.^10^ Childhood SLE showed fever (56.2%), rash (87%), arthritis (87%), renal involvement (56.2%), anemia (31.2%), thrombocytopenia (18.6%), Raynaud’s phenomenon (12.5%) and cardiac involvement (1%).^11^

Intraorally patient may present with ulcers, angular cheilitis, mucositis, glossitis, glossodynia, dysgeusia and dysphagia.^2^

Autoantibodies found in SLE are ANA, antinative DNA, rheumatoid factor, anti-Sm, anti-Ro and anti-La.^1^

Histopathologically, lichen planus and SLE are same. Karjalainen and Tomich described five histologic criteria to distinguish both.

1. Vascularization of keratinocytes
2. Subepithelial presence of patchy PAS positive deposits
3. Edema in upper lamina propria
4. PAS-positive thickening of blood vessel walls
5. Severe deep or perivascular inflammatory infiltrate.^12^

SLE is a chronic disease that waxes and wanes. Treatment should be focused on medication to control disease and measures to prevent flares. SLE can be treated with high-potency topical corticosteroids or intralesional steroid injections, cytotoxic drugs and plasmapheresis.^13^

SLE has an unpredictable history and course. Recognition and management of lupus flares and medication can markedly reduce morbidity and mortality allowing patients to live more functional lives.
